# British Medical Association

**Published:** 1895-08-10

**Authors:** 


### BRITISH MEDICAL ASSOCIATION.

Annual Museum.—(*Second Notice*).

Owing to the pressure on our columns last week we were compelled to hold over notices of some of the exhibits. We regret that owing to a printer's error the exhibit of Messrs. Willows, Francis, and Butler was described in our previous issue as that of Messrs. Parke, Davis, and Co. The animal extracts, &c., are prepared by the former firm, and not, as erroneously stated, by Messrs. Parke, Davis, and Co. Amongst the exhibits of the latter firm we noticed Taka-Diastase, the most powerful ferment for digesting amylaceous substances yet discovered. This substance, we are assured, converts 100 times its own weight of starch into sugar in ten minutes. Euthymol is an antiseptic preparation invaluable as a local application and for internal use.

Members of the profession visiting the exhibit of Messrs. C. J. Hewlett and Son will obtain some useful hints with regard to the economical stocking of their dispensaries. We noticed a cheap and serviceable ureameter for the rapid estimation of urea, and several useful clinical tests.

Messrs. Robinson and Sons (Limited) show a splendid assortment of antiseptic and absorbent dressings. They also exhibit some of the capital contrivances invented by Messrs. Reynolds and Branson for keeping bandages, pill-boxes, &c., in orderly and accessible form. Their patent waterproof accouchement and operation sheets are well worth inspection.

An enormous variety of abdominal belts are on view at the exhibit of the "Domen" Belts Company. The "Domen" varicose eirclet is intended to replace the clumsy and hot elastic stocking.

"Camwal" are well to the front with their aerated waters, which are manufactured in accordance with the British

Pharmacopœia for medicinal purposes in syphons and bottles.

Salamon and Co. (Limited) show samples of chloroform, the great features of which are its exceptional purity, its possession of a pleasant odour, and that it can be easily inhaled.

Messrs. Savory and Moore have as usual a number of new preparations. Their pancreatic emulsion provides the natural fat of food in a form in which it can at once be assimilated by the lacteals, and for use in rectal feeding it has obvious advantages; so, too, in cases of impaired digestion. Their fluid meat and their gelatine discs for ophthalmic and hypodermic purposes should not be passed by without notice.

Visitors to the museum have an opportunity of inspecting at the stall of Messrs. Duncan, Flockhart, and Co. the new "Baumol Soap," which has already attracted some attention in dermatological circles. It is a soap which is superfatted to the extent of 3 per cent., and constitutes a magnificent toilet article. Combined with carbolic or coal-tar preparations it becomes an excellent soap for antiseptic purposes.

As is well known, Messrs. Southall Bros. and Barclay are specialists in the preparation of concentrated tinctures, which, owing to the careful standardisation, can be relied upon as of constant strength. Among their novelties we may mention the granular effervescing citrate of colchicine, an elegant form in which to prescribe that remedy for acute gout, whereas the chronic form may equally well be treated by the New Aërated Lysidene Water, made according to Dr. Saundby's method. Their show of "chemists' sundries" is one of the finest in the museum, and their sanitary towels and specialities need no praise from us.

G. and G. Stern.—Pumiline is the speciality of this firm, and visitors are afforded an opportunity of inspecting it in the form of liniment, essence, or ointment—all of them excellent preparations. Narissa is a new food for invalids and children, and the Nura breast shields appear to offer many advantages.

Lacto-Peptine, which is shown by Mr. J. M. Richards, is too well known to require detailed mention.

Mr. B. Kuhn shows several preparations of Papain, now largely used as a supplementary digestive agent. Papier-Balmé is an ingenious form in which sublimate may be conveniently carried. One paper dipped in a pint of water forms a 1 in 1,250 solution. The Ethyl Chloride Bulb, a well-known means of obtaining local anæsthesia, is now provided with a curved as well as with a straight neck.

Terrol, the new tasteless substitute for cod-liver oil, is a liquid hydrocarbon, said to have high nutritive qualities, and of great use after influenza and other wasting diseases.

An attractive book-case is shown by Mr. Young J. Pentland, upon which, amongst other works, we specially notice "Diseases of the Joints," by W. Watson Cheyne; "Handbook of Obstetric Nursing," by F. W. N. Haultain and T. Haig Ferguson. The Compend Series contains a number of useful handbooks to assist students preparing for examinations, comprising those of medicine, surgery, obstetrics, gynæcology, pathology, and diseases of children.

Peptenzyme is a compound digestive agent, containing ferments from the different glands situated in the alimentary tract.

Samples of Stower's Lime Juice Cordial are shown by Messrs. A. Riddle and Co. This well-known table drink is invaluable to dyspeptic dispositions, and can be used in rheumatism, gout, fever, and inflammatory cases.

R. W. Greeff and Co. show a number of new preparations at present less generally known than they deserve to be. Gallanol is a substitute for pyrogallic acid. Salipyrin, a new salt of antipyrin and pirol, an odourless substitute for ichthyol.

Messrs. Lorimer and Co. are now introducing a new agen for the treatment of phthisis and other chest complaints; it is called Ethyl Phosphinate of Pyridin. It is reputed to be internally antiseptic. Vin Cocæ is a fine preparation of erythrozylon coca.

Messrs. Francis Newbery and Sons, as agents for William R. Warner and Co., show many of their excellent preparations, amongst which we were particularly impressed with the effervescing salts of various kinds, which are prepared in most convenient and portable form.

We must not overlook the Petroleum Emulsion of the Angier Chemical Company, which is gaining a good reputation as a gastric sedative. Horlick's Malted Milk has already won favour as a fine food for infants and invalids.

One of the features of Messrs Kutnow and Co.'s improved effervescent Carlsbad powder is its great adaptability for use in cases of infantile constipation. Those entrusted with the care of asthmatic cases will hail with pleasure the introduction of their new anti-asthmatic powder and cigarettes.

Mrs. A. M. Douglas shows an ingenious attachment to bedsteads, which by the aid of rollers and straps at the head and foot of the bedstead enables the sheet to be withdrawn without the patient being touched. By its aid the sheet can also be raised at either or both ends. Special sheets are, however, required for use with the attachment.

A visit to the Savoy Hotel next door was a natural termination to our round of the museum. In the "Victoria" rooms Messrs. Oppenheimer, Son, and Co. had on view a most interesting collection of medical and surgical antiquities, made by Dr. Luigi Sambon, of Rome, which well repaid a careful inspection. From thence we passed to the "Princess Ida" rooms, where Messrs. Hertz and Collingwood had, by special invitation, provided a sumptuous luncheon for members of the profession, with which was served their excellent and well-known Laurent Perrier "Sans-sucre" champagne. Samples of their Laurent Perrier "Coca-tonic" champagne (which we noticed at length in a recent issue) were also available, as well as of the new wine "Jerezcona," a fine sherry, tempered with cinchona, which this firm have just put on the market. We understand that some hundreds of members of the profession visited these pleasant rooms during the course of the exhibition, and partook of Messrs. Hertz and Collingwood's hospitality.

